# Left bundle branch area pacing in a patient with dextrocardia during torsades de pointes owing to Takotsubo syndrome

**DOI:** 10.1016/j.hrcr.2026.03.014

**Published:** 2026-03-24

**Authors:** Masaki Hashimoto, Kenichiro Yamagata, Masataka Yamasaki, Norihiko Takeda

**Affiliations:** Department of Cardiovascular Medicine, Graduate School of Medicine, The University of Tokyo, Tokyo, Japan

**Keywords:** Dextrocardia, Pacemaker, Left bundle branch area pacing, Takotsubo syndrome, Sick sinus syndrome, QT interval prolongation, Torsades de pointes

**Key Teaching Points**

• Dextrocardia is a rare congenital condition with abnormal cardiac anatomy, and there is no established method for pacemaker implantation, particularly for conduction system pacing.
• Takotsubo syndrome (TTS) causes transient cardiac dysfunction, which may sometimes persist. Patients with TTS and preexisting bradycardia may be suitable candidates for conduction system pacing, which also facilitates reliable follow-up of ST–T changes.
• Left bundle branch area pacing is a viable conduction system pacing method, and its application in patients with abnormal cardiac anatomy warrants further evaluation.

## Introduction

Right ventricular apical pacing has traditionally been considered the standard site for cardiac pacing. However, this approach carries the risk of long-term left ventricular dysfunction and may obscure electrocardiographic (ECG) signs of myocardial ischemia owing to pacing-induced left bundle branch block. Left bundle branch area pacing (LBBAP) may mitigate these concerns by using the native conduction system. Congenital anatomic irregularities make LBBAP challenging, given that it typically relies on a preshaped delivery catheter designed for normal cardiac anatomy. Therefore, reports of LBBAP in patients with dextrocardia—particularly those with situs inversus, in whom the heart is a mirror image of the normal configuration—remain limited. We present a case of successful LBBAP lead implantation in a patient with sick sinus syndrome (SSS) and dextrocardia with situs inversus, complicated by ongoing Takotsubo syndrome (TTS) and torsades de pointes (TdP), using a manually reshaped delivery catheter.

## Case report

**Figure 1:**
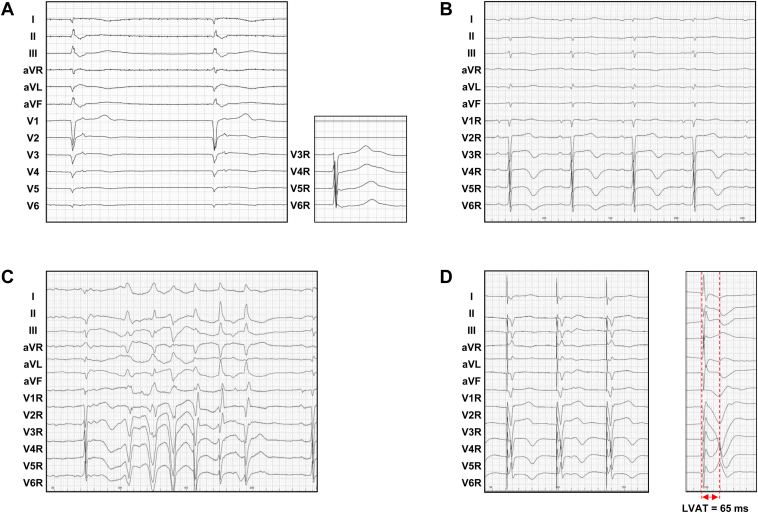
**A:** ECG 3 months before admission, showing sick sinus syndrome with junctional rhythm. **B:** ECG on admission with giant negative T wave and a QT prolongation. **C:** Torsades de pointes recorded on ECG during pacemaker implantation. D: ECG showing QRS morphology after left bundle branch area pacing. QRS duration of 122 ms with late R on V1R and left ventricular activation time of 65 ms. V1R to V6R indicates precordial leads placed on the right side of the chest in the mirror image of the normal position. ECG = electrocardiogram.

A 66-year-old man with dextrocardia and situs inversus was diagnosed as having SSS (Figure 1A) and heart failure, with an elevated B-type natriuretic peptide level and dyspnea on exertion when climbing stairs (New York Heart Association functional class II), and was indicated for permanent pacemaker implantation, although he hesitated for personal reasons. Transthoracic echocardiography demonstrated a preserved left ventricular ejection fraction of 67% with normal chamber dimensions. During follow-up, he experienced a sudden loss of consciousness and was transported to our hospital. 1 week earlier, he had a dispute at work. 2 days later, he had transient chest pain that resolved shortly afterward. The ECG on arrival showed a heart rate of approximately 30–50 beats/min, with giant negative T waves and a prolonged QT interval (Figure 1B). Transthoracic echocardiography revealed a left ventricular ejection fraction of approximately 45% with newly detected apical hypokinesis, leading to a diagnosis of SSS complicated by TTS. The ECG monitor repeatedly showed frequent episodes of TdP (Figure 1C). As bradycardia triggered TdP, the patient agreed to undergo permanent pacemaker implantation.

### Lead implant procedure

**Figure 2:**
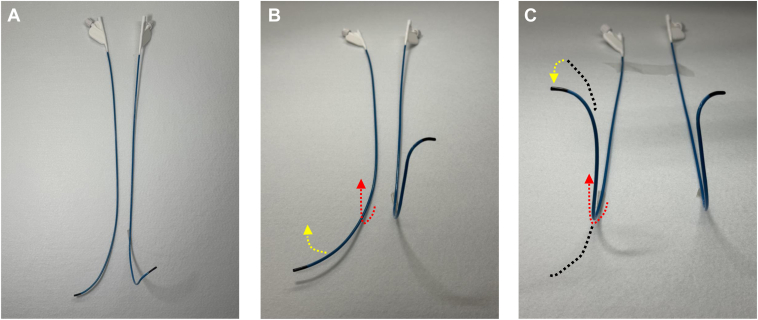
**A:** Native C315H40 catheter (left) and C315HIS catheter (right). **B:** Intended shaping of C315H40 catheter toward right ventricular septum in dextrocardia; bending points indicated by *red* (proximal) and *yellow* (distal) *dashed arrows*. **C:** Reshaped C315H40 catheter (left) and native C315HIS catheter (right).

**Figure 3:**
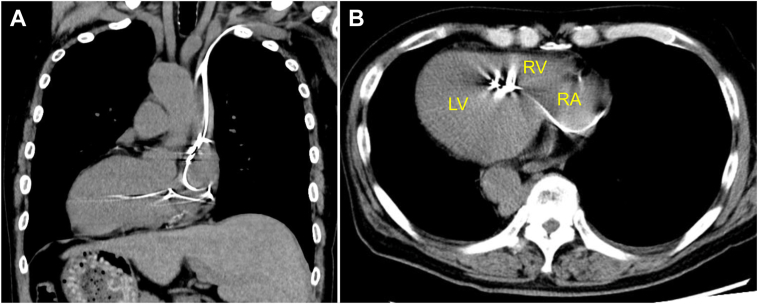
Computed tomography images showing atrial and ventricular leads after the operation. Coronal (**A**) and axial (**B**) computed tomography demonstrate the morphologic cardiac chambers in dextrocardia. Atrial and ventricular pacing leads are appropriately positioned within their corresponding morphologic chambers. LV = morphologic left ventricle; RA = morphologic right atrium; RV = morphologic right ventricle.

The procedure was performed under local anesthesia, and the precordial ECG leads were set for inversion of the heart, which is shown as V1R–V6R during the procedure. After making a pacemaker pocket on the left chest and inserting the sheaths, we initially implanted an atrial lead to resolve the sinus bradycardia-induced TdP. Thereafter, we reshaped the C315H40 catheter (Medtronic, Minneapolis, MN) to make the mirror image of the C315HIS (Medtronic), which is the standard catheter for LBBAP lead implantation (Figure 2). After obtaining a good unipolar pacing morphology on the ECG, the lumenless lead (3830 SelectSecure; Medtronic) was gently advanced and achieved successive LBBAP. The target site for ventricular lead insertion was determined using the 9-partition method under left anterior oblique fluoroscopic view.^1^ The vertical depth of lead advancement was assessed under the right anterior oblique view. Because the present patient had mirror-image dextrocardia, the fluoroscopic orientation of right anterior oblique and left anterior oblique views was reversed compared with the usual cardiac anatomy. Adequate depth was confirmed by posterior migration of the R wave in lead V1, resulting in a terminal R pattern.^2^ The QRS duration was 122 ms, and the left ventricular activation time in lead V6R was 65 ms (Figure 1D), indicating successful LBBAP.^2^ Capture threshold, sensing amplitude, and lead impedance were all within acceptable ranges. Giant negative T wave was apparent on the ECG, remaining unchanged from that observed before LBBAP initiation (Figure 1B and 1D). After the procedure, the computed tomography showed that the lead was implanted in the morphologic right ventricular septum (Figure 3). The B-type natriuretic peptide level decreased from 403 pg/mL before the procedure to 19 pg/mL at 10 days postoperatively, and the patient’s symptoms resolved. The patient was discharged on the 12th postoperative day after normalization of cardiac function, without any device-related complications.

## Discussion

Dextrocardia is a rare congenital condition with an incidence of approximately 1 in 12,000 births.^3^ Dextrocardia can be subdivided into 3 types: (1) dextrocardia with situs solitus, where the heart is located on the right side but other organs are normally positioned; (2) dextrocardia with situs inversus, where both the heart and all other organs are mirrored; and (3) dextrocardia with situs ambiguous, where the positions of the organs are not clearly defined, leading to anatomic complexity.^4^ Intracardiac procedures are particularly challenging in patients with dextrocardia owing to abnormal cardiac anatomy. Although several cases have been reported, there is no established method for pacemaker implantation in patients with dextrocardia.^5^^,^^6^ This is a case report of a patient with dextrocardia and situs inversus, complicated by SSS and TTS, which caused TdP. For patients with SSS, atrial pacing using a single-chamber pacemaker in AAI mode is often sufficient; however, a dual-chamber pacemaker may be selected to account for the possibility of future atrioventricular node dysfunction. Notably, atrioventricular node disturbances can remain problematic in TTS, even in the chronic phase.^7^^,^^8^ Given that some patients with TTS continue to exhibit cardiac dysfunction into the chronic (or remote) phase,^9^^,^^10^ we performed conduction system pacing in the current patient. LBBAP is a method of conduction system pacing that has been increasingly used in recent years and requires a preshaped delivery catheter to capture the left bundle branch area. To achieve LBBAP, the ventricular lead is implanted on the right ventricular septum, approximately 1–2 cm distal to the His bundle area, using a delivery catheter. The C315HIS delivery catheter is naturally shaped to direct the lead toward the ventricular septum in patients with normal cardiac anatomy; however, for patients with dextrocardia, manual reshaping of the catheter is required, as previously reported.^11^, ^12^, ^13^ However, previous reports required more aggressive reshaping of the sheath to reverse the original 3-dimensional curve designed for normal heart anatomy. Our procedure for reshaping the C315H40 does not require these aggressive reshaping techniques, given that it uses the original curve of the catheter and enables adding a curve according to the right atrial size, which is usually enlarged in patients with congenital heart disease. In the current case, we confirmed under fluoroscopy that the lead was in contact with the interventricular septum by observing the movements of the lead and sheath and considering action–reaction forces; however, contrast injection via the sheath is useful, and imaging tools such as transthoracic echocardiography, transesophageal echocardiography, and intracardiac echocardiography may also help identify the interventricular septum in complicated cases.^14^, ^15^, ^16^, ^17^ Several cases of LBBAP in patients with dextrocardia have been reported; however, to the best of our knowledge, this is the first case report of successful LBBAP lead implantation in a patient with SSS and dextrocardia with situs inversus, complicated by TTS and TdP, where ST–T morphology assessment is important. Right ventricular pacing typically obscures the ST–T abnormalities owing to secondary repolarization changes.^18^ In our case, ST–T morphology remained unchanged with LBBAP initiation. Although previous reports describe detection of acute coronary syndrome–related ST–T changes in patients with LBBAP, none have shown reproducible pre- and postpacing morphology in the acute phase.^19^, ^20^, ^21^ This case indicates that LBBAP may facilitate reliable ST–T monitoring in these situations.

## Conclusion

We present a case of SSS with dextrocardia and situs inversus, complicated by TTS and TdP, in which an LBBAP lead was successfully implanted. Currently, no standardized implantation protocol exists, and the long-term outcomes of LBBAP remain unclear, particularly in patients with dextrocardia. Therefore, a standardized implantation protocol for LBBAP in patients with abnormal anatomy, such as those with dextrocardia, should be established as more long-term outcome data become available.
